# Synthesis of Biomaterials Utilizing Microfluidic Technology

**DOI:** 10.3390/genes9060283

**Published:** 2018-06-05

**Authors:** Xiaohong Wang, Jinfeng Liu, Peizhou Wang, Andrew deMello, Lingyan Feng, Xiaoli Zhu, Weijia Wen, Rimantas Kodzius, Xiuqing Gong

**Affiliations:** 1 Materials Genome Institute, Shanghai University, Shanghai 201800, China; wxh0201@t.shu.edu.cn (X.W.); Perpethere@shu.edu.cn (J.L.); lingyanfeng@t.shu.edu.cn (L.F.); phwen@ust.hk (W.W.); 2 Advanced Placement of Chemistry Program, International Department, Huzhou New Century Foreign Language School, Huzhou 313100, China; xiey2@uci.edu; 3 ETH Zurich, Zurich 8093, Switzerland; andrew.demello@chem.ethz.ch; 4 School of Life Sciences, Shanghai University, Shanghai 200444, China; xiaolizhu@shu.edu.cn; 5 Mathematics and Natural Sciences Department, the American University of Iraq, Sulaimani, Sulaymaniyah 46001, Iraq; 6 Faculty of Medicine, Ludwig Maximilian University of Munich (LMU), 80539 Munich, Germany; 7 Faculty of Medicine, Technical University of Munich (TUM), 81675 Munich, Germany

**Keywords:** microfluidics, biomaterials, microparticles, microfibers, liposomes, artificial cells, tissue engineering

## Abstract

Recently, microfluidic technologies have attracted an enormous amount of interest as potential new tools for a large range of applications including materials synthesis, chemical and biological detection, drug delivery and screening, point-of-care diagnostics, and in-the-field analysis. Their ability to handle extremely small volumes of fluids is accompanied by additional benefits, most notably, rapid and efficient mass and heat transfer. In addition, reactions performed within microfluidic systems are highly controlled, meaning that many advanced materials, with uniform and bespoke properties, can be synthesized in a direct and rapid manner. In this review, we discuss the utility of microfluidic systems in the synthesis of materials for a variety of biological applications. Such materials include microparticles or microcapsules for drug delivery, nanoscale materials for medicine or cellular assays, and micro- or nanofibers for tissue engineering.

## 1. Introduction 

Having emerged in the beginning of the 1980s, microfluidics is a highly interdisciplinary science and technology of systems that process or manipulate small (9–10 to 10–18 L) amounts of fluids, using channels with dimensions of tens to hundreds of micrometres. It incorporates knowledge and techniques that intersect physics, chemistry, mechanics, nanoscience, and biotechnology, with practical applications in the design of systems in which small volumes of fluids are well-controlled [1,2]. As part of the vast field of microfluidic application, the role of the microfluidic chip in synthetic chemistry and biology creates a new instrumental platform that is the microfluidic reaction system. It deals with the material synthesis by the flow of a minute amount of liquid (which can be ≈5–9 orders of magnitude smaller than those associated with bench-top chemistry) and within micrometer-sized channels [3,4]. These features offer clear advantages over bulk synthesis, most notably in their ability to fine-tune the physical properties and chemical compositions of the final product. Meanwhile, they can provide an efficient way with which to control mixing, save sample consumption, obtain uniform particle size, and create materials with novel structures and functions [5]. Although microfluidic technology has been applied to prepare of diverse range of materials, for example, micro- or nanoparticles, microgels, micelles or vesicles, organic molecules, etc., this review only aims to provide an overview of the recent methods and techniques that have been designed in synthetic biology in the field of bioengineering and biomedicine, which include liposomes for artificial cells or organelles, micro-/nanofibers for tissue engineering, and micro-/nanoparticles for drug delivery. Novel bioengineering and biomedicine materials are always necessary to meet the increasing demand for a comprehensive understanding of the operating mechanisms of organisms and an effective way to treat disease. It has been confirmed that microfluidic technology provides a new pathway with which to exploit biomaterials with novel properties and functions.

Indeed, there is a recognized desire to build artificial cells and tissues to accelerate the study of the basic biological reactions of life in a bottom-up manner. Put simply, chemists and biologists aim to prepare a variety of biological materials (such as lipids, proteins, nucleic acids, and glycans) to study the fundamental building blocks of life.

It is widely accepted that cells are the minimal units of life and are required to form complex organisms. Lipid vesicles or liposomes can in principle be prepared by the self-assembly of amphiphilic molecules to mimic the role of organelles or cells and provide well-defined compartments for the performance of processes such as gene expression, enzymatic reactions, protein synthesis, material transport, and signal transmission. Additionally, in biomedical research, liposomes can be used to encapsulate cells for studying cells behavior or responses to drug stimulation. Microfibers, on the other hand, have been widely used as scaffold materials in tissue engineering applications. For example, encapsulating cells on microfibers provides a basic three-dimensional (3D) model for cell culture and complex tissue formation [6]. In biomedical applications, materials with special structures and properties have been fabricated to allow the smart release of drugs. Due to their unique physical structure, such materials can be used to prevent the clearance by immune cells and provide control of achievable release rates.

Although these materials have contributed to splendid developments in biomedicine and life science, there is a pressing need to develop flexible methods for their preparation in a robust, reproducible, high-throughput, and cost-effective manner. In this respect, the microfluidic reaction system has emerged as a promising tool in the high-throughput, controlled, and direct preparation of materials for the diversity of biological and chemical applications [7,8,9,10]. Due to the laminar flow environment that is typical of microfluidic channels, reactions can be performed in a highly controlled and reproducible manner. In addition, the size and composition of products can be flexibly designed through adjustment of the flow rate, feature geometries, and the nature of input reagents. Furthermore, unlike almost all conventional methods, microfluidic techniques allow for the continuous production of novel materials in a single workflow. Most importantly, many reactions within microfluidic environments may be performed under mild conditions—a feature that is highly beneficial for many bioactive reaction systems. Accordingly, these advantages have engendered many new initiatives in the field of synthetic biology. Plenty of materials for biological applications can be prepared using microfluidic methods, including nanoparticles for biomedical applications [11], microcapsules [12] and microparticles [13] for biomedical delivery and diagnostic applications, barcodes for multiplex assays [14], liposomes for building artificial cells and organelles [15], and micro- (or nano-) scale fibers for tissue engineering [16,17]. The following section provides a general overview of the state-of-the-art methods for preparing biomaterials via microfluidic techniques and highlights the use of liposomes, microfibers, nanoparticles/microparticles, microgels, and microcapsules in different biological applications.

## 2. Liposomes for Artificial Cell Systems and Organelles

Liposomes, a special type of vesicle, are usually composed of one or several shells made from amphiphilic molecules, such as natural and synthetic phospholipids. At a basic level, the self-assembly of phospholipid molecules in the aqueous phase, and driven by the hydrophobic effect, lead to the formation of liposomes, which have wide potential applications in pharmaceutical science, cosmetic and consumer industries, and the manufacture of artificial cells. Due to their biocompatibility and integrity, liposomes have been used to prepare and encapsulate number of active biomolecules, including enzymes, proteins, vaccines, hormones, and nanomaterials [18,19].

In a simple sense, there are four important features that control the utility of liposomes in biological applications: (1) Membrane composition and lamellarity. Membrane composition has significant influence on mechanical and chemical properties of liposomes; in addition, lamellarity of liposomes should be well-controlled for specific applications, for example, unilamellar liposomes are usually used in membrane protein studies while multilamellar liposomes are mainly used for controlled release system; (2) The average size and size distribution. Although different sized liposomes cater to different applications, monodisperse liposomes are highly desired in most applications that ensure the stability and dose of reagents encapsulated in the interior of liposomes; (3) Liposome stability. Liposomes stably stored last for long time and have exception flexibility in practical applications; (4) Encapsulation efficiency. High encapsulation efficiency is desired for most applications [20]. It is accepted that most conventional methods used to fabricate liposomes yield polydisperse liposomes populations and are characterized by low yields and encapsulation efficiencies. More specifically, most of these methods involve tedious post-processing to isolate liposomes with desirable properties. In recent years, microfluidic techniques have been used with good effect in the preparation of liposomes. They provide a confined microenvironment for liposome formation and have been successful in controlling liposome size, size distribution, and lamellarity.

Fluid flow within microfluidic channels is almost always laminar, with Reynolds numbers being significantly below 1. In this flow regime, inertial forces are dominated, and mixing between parallel streams is mainly dependent on passive molecular diffusion. Unlike the macroscale, mixing in microchannel is driven by the small diffusion length scale that improved diffusion and advection and achieved robust and efficient mixing [21]. Considerable efforts were contributed to the design of microdevices with delicate structures to further enhance the mixing in microchannel. According to the mechanism employed to induce mixing within microscale, micromixers mainly include two types: one is active micromixers that need external power such as electricity and pressure field, and the other is passive micromixers in which mixing is improved by special microchannel structures. Therefore, microfluidic reactors have many appealing characteristics for preparation of nanomaterials and micromaterials, including efficient and fast mass and heat transfer, precise control of reaction, highly integrated process under continuous flow condition, and potential of large-scale engineering industry. Providing homogeneous and highly-controlled conditions is prerequisite to prepare liposomes with superior physical and chemical properties. Microfluidics technique with above advantages offers an ideal environment for fabricate liposome with uniform size and designable structures.

There are number of excellent review articles assessing the use of microfluidic devices in the preparation of liposomes [15,20,22]. A variety of microfluidic methods have been used to fabricate liposomes with great uniformity, including techniques based on electroformation, hydration, extrusion, flow focusing, microfluidic jetting, double emulsion templates, and phase transfer. It should be noted that electroformation and hydration methods are the most popular conventional routes for liposome preparation [23]. However, liposome populations made using both methods are characterized by wide size distributions, which significantly limits their applications in biomedicine. For example, Kuribayashi et al. successfully combined microfluidics with electroformation to form liposomes [24]. Although reactions were observed to be faster, liposome size distributions remained unacceptably high. The size uniformity of liposomes was improved by changing the surface properties of the substrate [25]. Recently, in order to achieve a cost-effective production of liposomes, stainless steel electrodes were employed to make giant unilamellar vesicles (GUVs) in a rapid and scalable electroformation manner [26]; additionally, GUVs were formed on indium tin oxide (ITO) electrodes in saline solution and from charged liposomes under physiological conditions [27]. However, this method requires extra equipment to supply electricity and has limited ability to adjust composition and structure of liposomes. Indeed, preparation methods based on different microfluidic techniques are characterized by specific advantages and disadvantages on a case-by-case basis. According to the practical application, we choose the suitable method to prepare liposomes. For example, the encapsulation efficiency is less important when liposomes are employed to study membrane protein; thus, electroformation and extrusion are suitable methods. While encapsulation efficiency is considerably significant for liposomes as a cell model or drugs delivery system, droplet emulsion technique is more suitable than electroformation. In a word, according to different applications, you have different choice. Table 1 shows some of the most important advantages and disadvantages of different microfluidic methods for liposome fabrication.

### 2.1. Microfluidic Hydrodynamic Focusing for Liposomes Production

Microfluidic hydrodynamic focusing (MHF) is popular amongst microfluidic methods to fabricate nanoscale liposomes or vesicles, because it can provide high-throughput and continuous production of monodisperse liposomes without multiple post-processing steps. The physical and chemical properties of liposomes prepared in this way are flexible and tunable, with liposomes or vesicles diameters on the nanometer scale.

**Figure 1:**
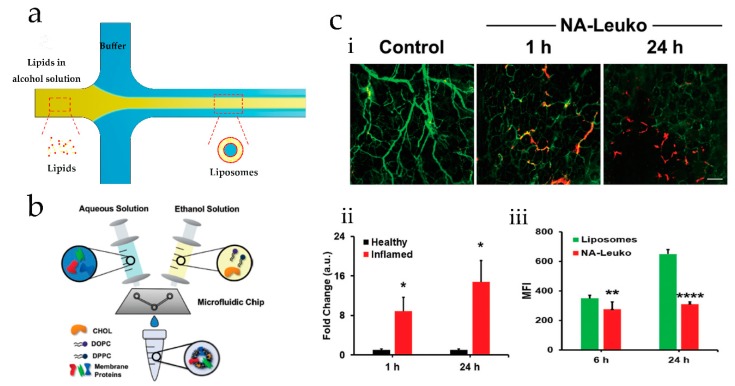
Microfluidic hydrodynamic focusing (MHF) methods used to prepare liposomes or vesicles. (**a**) Schematic of an MHF device used to prepare liposomes; (**b**,**c**) Biomimetic nanovesicles were prepared by the MHF method. (**b**) Schematic diagram of device, (**c**) in vitro and in vivo biological properties of liposome-like nanovesicles (NA-Leuko), (**i**) in vivo inflammatory targeting of NA-Leuko in localized ear inflammation mold, (**ii**) quantification of NA-Leuko, into the inflamed ear compared to healthy ears at different time after injecting, (**iii**) consequence of flow cytometry (6 and 24 h incubation) in vitro uptake studies of control liposomes and murine NA-Leuko following incubation with J774 macrophages (reprinted with permission from [36]).

Typically, an alcohol solution containing dissolved lipids flows through a center inlet and meets an aqueous solution flowing through the two side channels at a cross-junction (Figure 1a). This allows a well-defined and predictable interface between the streams to form, which results in the spontaneous self-assembly of lipids into closed spherical liposomes [30]. The size and size distribution of resulting liposomes can be simply tuned by adjusting the flow rate ratio (FRR) and total flow rate (TFR). Using such an approach, Kastner et al. found that the FRR has more impact on liposome size and size distribution than TFR [32]. Additionally, the authors found that the FRR could be used to control transfection efficiency in vitro, whilst the TFR could not. Moreover, data analysis indicated that transfection efficiency could be improved by increasing liposomes size.

The formation of size-predictable liposomes through spontaneous self-assembly is dependent on properly understanding the nature of mixing. In this regard, Phapal et al. compared the impact of mixing strategy (bulk injection, stream mixing, and laminar microfluidic mixing) on the formation of liposomes from a lipid/ethanol solution and an aqueous solution [33]. The authors found that only the two former methods indicated that liposomes properties were dependent on the lipid concentration in ethanol. This could be understood by assuming that liposomes size is strongly correlated with the nature of microconvective mixing. In related studies, Hood et al. argued that the wetting of mixing microchannels using the ethanol solution might be a significant cause of polydispersity [34]. Accordingly, the authors designed and tested a coaxial 3D MHF device for the preparation of nanoscale liposomes, with products displaying a high level of uniformity. To enhance the throughput of liposome fabrication, Hood and DeVoe subsequently designed a microfluidic vertical flow focusing (VFF) device containing high aspect ratio microchannels [35]. Results showed that VFF microfluidic devices not only afforded high-throughput liposome production (1.6 mg/min) but also maintained low polydispersity and high stability of produced liposome populations.

In consideration of applications in drug delivery, prepared liposomes should be characterized by adequate stabilities, high encapsulation efficiencies, and stable drug release profiles. In work by Correia et al., it was shown that liposomes manufactured by MHF were stable over a period of three weeks and exhibited high encapsulation efficiencies [37]. Additionally, the drug interactions with liposomes and release rates could be controlled by adjusting the operating conditions. In some target delivery systems, it is important to be able to modify the surface properties of liposomes, which in turn allows therapeutic materials to be recognized by target cells or tissues, whilst avoiding clearance by immune cells. For example, polyethylene glycol and folic acid have been successfully incorporated into liposomes prepared using microfluidic reactors [38,39]. In these studies, modified liposomes achieved efficient cellular uptake both in two-dimensional (2D) cell culture models and 3D tumor spheroids.

Recently, membrane proteins derived from leukocytes were successfully incorporated into the lipid bilayer of liposome-like nanovesicles (NA-Leuko) using a microfluidic device called the NanoAssemblr (NA) [36]. This device consists of two sections, namely, a Y-type inlet channel and a “herringbone” mixing channel (Figure 1b). Using this microfluidic platform, liposomes with a mean diameter of 118 nm and low polydispersity could be continuously prepared. Moreover, the incorporation of membrane proteins resulted in an increase of bilayer rigidity but did not lead to adverse effects on the stability of the nanovesicles. These nanovesicles performed excellent selective inflamed targeting properties to inflamed ear while displaying no difference in accumulation towards non-inflamed ears after 24 h (Figure 1ci,cii). When incubated with syngeneic macrophages, they showed a considerable reduction in uptake when compared to control liposomes (Figure 1ciii). All these observations indicated that NA-Leuko vesicles produced in a microfluidic reactor have a promising prospect in drug targeting/delivery systems.

The MHF method offers an exquisite way to prepare nanoscale liposomes or functional liposomes with low levels of dispersity in a robust, simple, and reproducible manner. Although this technique, to some extent, allows high-throughput fabrication of liposomes with controlled properties, it has yet to be transferred to an industrial environment for large-scale liposome production.

### 2.2. Droplet Emulsion-Based Technologies for the Generation of Artificial Cells

Droplet-based microfluidic systems all direct production of uniform droplets, which can be used as templates for liposomes production. In droplet-based microfluidics, the flow containing at least two liquids was controlled by volume or pressure. Droplets were generated by surface induced instability using different device geometries, including T-junction and flow-focusing. For preparation of liposomes, two liquids were introduced into droplet-based microfluidics system, oil phase containing lipid as continuous phase, and aqueous phase as dispersed phase. Lipid-stabilized droplets were generated in the interface of continuous oil phase and dispersed aqueous phase. Double emulsion templating is a highly popular method for the fabrication of uniform liposomes or GUVs with bilayer membrane. The size and the encapsulation efficiency of liposomes can be easily controlled by tuning the flow rate and the concentration ratio of aqueous and oil phases. Additionally, cells, biologically active molecules such as proteins, and some functional materials can be controllably encapsulated into liposomes [40].

The phase transition method (based on droplet emulsification) is popular for preparing GUVs in a rapid and efficient manner. For example, Paegel et al. designed a simple microfluidic assembly line for generation of unilamellar vesicles [41]. First, a lipid-stabilized droplet with an aqueous core is produced in a flow-focusing region. Subsequently, the droplet phase transfers via a triangular guide after flowing in an aqueous/oil co-flow with a stable oil/water interface. Finally, these droplets were transferred to vesicles with intact bilayer membrane. Vesicles produced in this manner are highly uniform and possess diameters between 20 and 70 μm. 

**Figure 2:**
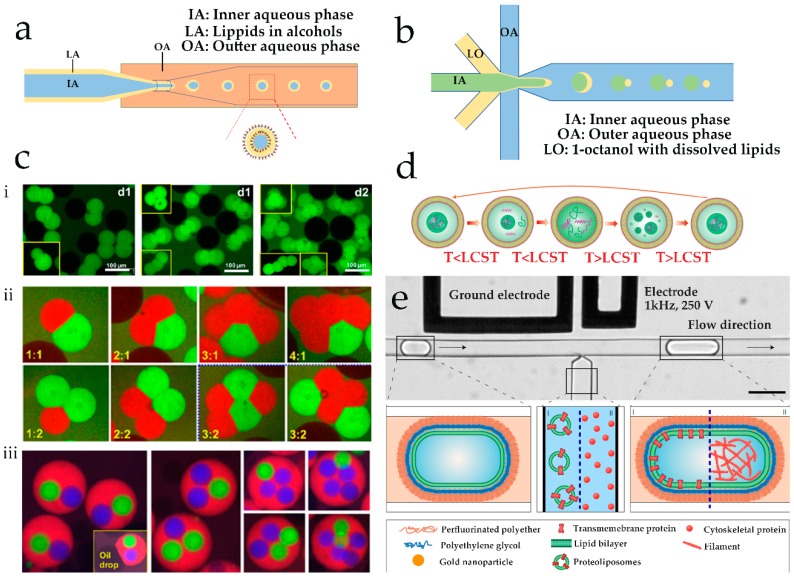
Droplet emulsion-based method used to fabricate liposomes. (**a**) Schematic of a microfluidic double emulsion process; (**b**) an octanol-assisted liposome assembly (OLA) in a microfluidic device; (**c**) liposomes with various structures prepared by microfluidic double emulsions; (**i**,**ii**) multicompartment liposomes with uniform (**i**) or distinct components (**ii**) (reprinted with permission from [43] Copyright (2016) American Chemical Society); (**iii**) vesosomes with different number of liposomes in their core (reprinted with permission from [44] Copyright (2017) American Chemical Society); (**d**) thermal dynamics of the membrane-less organelle-like compartment in liposomes (reprinted with permission from [45]); and (**e**) schematic process for incorporating biomolecules into droplet-stabilized giant unilamellar vesicles (dsGUVs) through pico-injection droplet microfluidic technology, scale bar: 50 μm (reprinted with permission from [46]).

Combination of a double-emulsion template microfluidic method and solvent-extraction allows liposomes and polymersomes with low levels of dispersity to be fabricated in a continuous and controlled manner [31]. In fact, a significant disadvantage associated with the droplet emulsion method for preparing liposomes or vesicles is the presence of residual solvent in the bilayer (Figure 2a). Although Karamdad et al. showed that the rigidity of the membrane in prepared vesicles was not especially susceptible to the presence of residual oil in the bilayer [42], residual solvent in the bilayer may affect the behavior of protein modified on the membrane or membrane permeability. An octanol-assisted liposome assembly (OLA) in a microfluidic device designed by Deshpande et al. enabled the separation of residual solvent in the bilayer and unilamellar liposomes [42] (Figure 2b). The authors found that when using 1-octanol as the lipid-carrying organic phase, the OLA process was both faster and biocompatible when compared to existing methods. Unilamellar liposomes are cell-sized (with diameters between 5 and 20 μm), exhibit high encapsulation efficiencies and uniformity, and have promising potential as nanoreactors for functional protein expression.

Based on this method utilized by Deshpande et al. [42], Deng et al. presented a surfactant-assisted method to assemble liposomes based on microfluidic double emulsions [43]. Pluronic F-68, a triblock copolymer surfactant, was used to tune interfacial energies in the emulsion system to manufacture solvent-free liposomes with single or multiple compartments. Through variation of surfactant concentration and template dimensions, liposomes with diameters between 20 and 200 μm can be produced from less than 1 min to about 3 h. In vitro transcription and translation in the prepared liposomes indicated that liposomes exhibited high feasibility in cell-free gene expression. Most importantly, liposomes with multiple compartments could be prepared using this method (Figure 2ci,cii), a process inaccessible to conventional methods. Subsequently, vesosomes, a special subset of liposomes encapsulating multilayered liposomal structures, were fabricated using the same system [44] (Figure 2ciii). The number of liposomes within a vesosome could be directly controlled through adjustment of volumetric flow rate. In addition, the volume of the shell between interior and exterior liposomes can be designed in a specific manner to meet the requirements of special delivery system. Furthermore, this method was successful at encapsulating different materials within different sub-compartments. For example, the authors inserted melittin, a membrane protein, into the liposome core to create nanopores, and achieved controlled transportation of nutrient molecules. Additionally, transcription and translation could be conducted in independent compartments of a vesosome. All these features confirm that the microfluidics method offers an excellent route with which to prepare artificial cells. Indeed, recently, the same team prepared cell-sized liposomes encapsulating coacervates as artificial, non-membrane-bound sub-compartments through combination of the microfluidic method with phase separation of oppositely charged polyelectrolytes [45]. The prepared coacervates are highly-ordered and located in the middle of liposomes with thermal-responsive reversible dynamics of coacervation and dissolution (Figure 2d).

Polymersomes prepared from amphiphilic block copolymers have been shown to provide alternative route towards artificial cells. However, the manipulation of the physicochemical properties of such structures is an ill-defined process. Recently, droplet-stabilized giant unilamellar vesicles (dsGUVs) have been prepared within automated microfluidic platform at high throughput [46]. The compartments in such dsGUVs possess a high level of mechanical and chemical stability, are cell-like in size, and can be controllably loaded with various biomolecules in sequential manner (whilst maintaining membrane integrity) through picoinjection methods (Figure 2e). Interestingly, the assembled lipid compartments could be released from the surrounding stabilized polymer droplets into physiological fluids to allow evaluation of their interaction with physiological environments. Significantly, results showed that the biofunctionality of biomolecules encapsulated in vesicles is not affected by the reconstitution and release process. Indeed, the described method provides a versatile and high-throughput route towards the generation of functional and bespoke dsGUVs based on the bottom-up assembly of intracellular modules.

### 2.3. Recent Efforts towards Cost-Effective Production of Liposomes in Microfluidic Systems

Recently, plenty efforts are contributed to design microfluidics devices for preparation of liposomes in a cost-effective manner. As far as we know, although microfluidics chips have numerous advantages to fabricate liposomes, there is a big challenge to develop this technique towards industry, because those microfluidics devices are usually manufactured by expensive equipment and clean room facilities. Therefore, creating a simple and cost-effective method for fabrication of microfluidics device will largely encourage this technology towards market. Petit et al*.* employed a polydimethylsiloxane (PDMS) microfluidics chips with a hydrophilisation of the external channel rather than complex modification to fabricate liposomes, which greatly make fabrication of microfluidics devices simple and reduce cost of device [31]. In addition, the liposomes prepared has high monodispersity and stability, which could be stored with 3 months under ambient conditions. Zhang et al. developed a 3D printed mould casting method for fabrication of PDMS based continuous-flow reactors [47]. Through this process, the PDMS microfluidics chip could be fabricated within 24 h and a low cost of 5 £. Most importantly, microfluidics chips demonstrated relatively comparable performance for preparation of liposomes and silver nanoparticles like those devices fabricated with expensive equipment. Nastruzzi et al. designed a simple “off-the-shell” microfluidic reactor to fabricate liposomes [48]. The liposomes prepared in these devices were unilamellar, and the preparation was highly reproducible. This result demonstrated this fabrication technique had potential common to prepare liposomes.

In summary, droplet-based microfluidic system has generated a great deal of excitement as a method of production of uniform liposomes with well-defined structures at a high frequency. Especially, the research of Deshpande et al. [42]. and Deng et al. [43,44]. demonstrated that GUVs without residual oil in the bilayer could be continuously fabricated through double-emulsion microfluidics techniques. For engineering artificial cells application, substantial research efforts should be directed towards the designation of liposomes with high-order compartmentalization for mimicking various living bioactions simultaneously processing within cell level in the future. In addition, liposomes as synthetic cells require a high chemical and mechanical stability under high ionic strength condition and require sequential and controllable loading and permeability of bio-molecules under different conditions; thus, it is highly expected to prompt exploration of preparation of liposomes with increased membrane complexity, which endows the liposomes even more flexibility and functionality.

## 3. Microfluidic Spinning of Micro-/Nanofibers for Tissue Engineering

Tissue engineering has received increasing attention due to its potential applications in regenerative medicine, replacement of damaged organs, and non-animal drug testing [49]. A key priority of tissue engineering research is to develop methods suited to the manufacture delicate scaffold materials to support the proliferation, alignment, and differentiation of cells. Indeed, it should be noted that most human organs or tissues maintain their basic morphology with the help of biological micro or nanofibers, such as collagen and elastin. Accordingly, much work has been done in preparing synthetic various microfibers for 3D cell culture and tissue reconstruction [6]. In general, there are several significant requirements for microfibers used as scaffolds. These include (1) a stable structure with good mechanical properties; (2) integrated microstructures for cell infiltration, adhesion and proliferation; and (3) non-toxic and biocompatible to physiological environment [16]. Unfortunately, most traditional methods used to fabricate fibers, such as melt spinning, wet spinning, and electrospinning [50,51], are unsuited to making fibers with multiple structures and morphologies. Moreover, another problem hindering their use in the preparation of fibers for biological applications is the need to use organic solvents.

Unlike conventional methods, microfluidic spinning shows more flexibility and reproducibility in the synthesis of fibers of uniform size and adjustable composition in ambient environments, a process inspired by the natural process of silk-spinning of spiders or silkworms [16,17,52]. Fibers with a variety structures can be produced, including solid cylinders, hollow tubes, flat fibers, Janus structures, spiral curls, and bamboo-like architectures using coaxial laminar flows. In addition, some natural polymers and hybrid fibers can be more easily prepared within microfluidic platforms in a continuous manner compared to some traditional spinning methods. Furthermore, microfluidic spinning has reduced sensitivity to operational parameters and thus exhibits a high degree of reproducibility. Generally, cross-linking of fibers involves photopolymerization, diffusion-controlled ionic cross-linking, and solvent extraction. Table 2 summarizes the features of those three methods in microfluidic spinning for preparation of microfibers. To this end, we now discuss recent developments in the use of microfluidic spinning to manufacture fibers according via crossing-linking.

### 3.1. Photopolymerization

**Figure 3:**
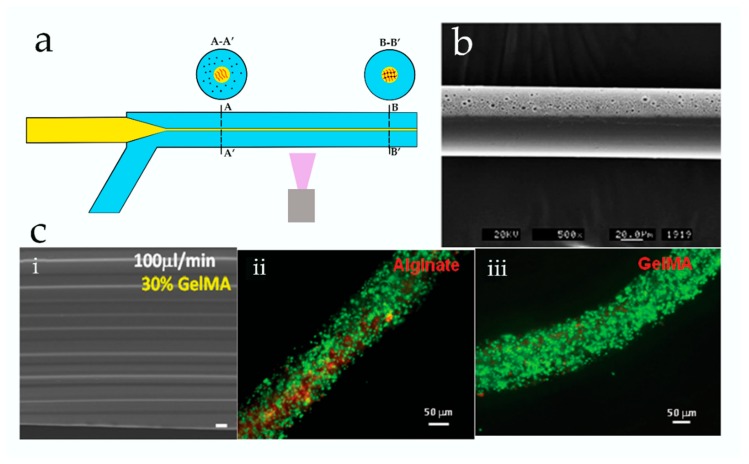
Photopolymerization combined with microfluidics spinning methods used to fabricate microfibers. (**a**) Three-dimensional microfluidic hydrodynamic device used to prepare solid microfibers with photopolymerization method; (**b**) Janus microfibers fabricated by photopolymerization in microfluidic device (reprinted with permission from [54]); (**c**) flat microfibers with a grooved surface prepared by microfluidic spinning; (**i**) scanning electron microscop image, scale bar 20 μm; (**ii**,**iii**) fluorescence images cultured on alginate fibers; (**ii**) and methacrylamide-modified gelatin (GelMA) fibers; (**iii**) (reprinted with permission from [55]).

Photopolymerization is a simple and direct method for the fabrication of microfibers. In brief, polymerization is induced by ultraviolet (UV) radiation (usually 365 nm) after pre-polymers are mixed with a photoinitiator. For example, Lee and co-workers reported “on-the-fly” polymerization of 4-hydroxybutyl acrylate (4-HBA) using a simple 3D microfluidic hydrodynamic device [53] (Figure 3a). The diameter of fibers could be flexibly adjusted through tuning the flow rate ratio between the sample and sheath flows. The resulting fibers exhibited good elasticity and a rapid response to changes in pH. At the same time, the authors successfully used the fibers as a sensitive glucose biosensor by immobilizing glucose reactive enzymes onto fibers. Since the polymerization of 4-HBA is fast, the activity of enzymes is not affected by UV radiation. However, the attachment of living cells to such fibers is yet to be reported.

NIH3T3 fibroblast cells were also successfully proliferated on Janus polyurethane (PU) microfibers prepared via in situ microfluidic photopolymerization [54] (Figure 3b). Janus PU fibers possess both a porous region and non-porous region, which results from release of CO_2_ bubbles formed during reaction. Preparation of porous fibers by conventional methods requires large quantities of organic solvents that are harmful for many biological applications. Interestingly, studies indicated that the NIH3T3 cells only adhered to the porous region and completely covered the entire surface of the Janus fibers after 120 h of culture. Additionally, cell sheets or aggregates were formed through connection with a long cell bridge about 200 μm. This observation has some significance for studies assessing cell performance in 3D scaffolds and in tissue regeneration.

To avoid exposure to UV light, cells can be constrained as a core flow surrounded by pre-polymers that polymerize on UV radiation and provide a protective shell [75]. Indeed, a specially designed microchannel integrating with grooves has been used to direct a poly (ethylene glycol dimethacrylate) (PEGDMA) pre-polymersolution mixed with the cells towards the center of microchannel, a key aspect in the formation of core-shell structures. Metabolic activity and growth assays indicated that over 90% cells encapsulated survive polymerization and retain their metabolic activity, which represents a considerable improvement compared to electrospinning methods (which are characterized by survival rates below 50%).

When considering tissue reconstruction, cell alignment is an important property for microfibers when acting as scaffold materials. Generally, some biological functionalities can be added to the surface of fibers to induce cell adhesion; however, these reactions are labor-intensive and the functionalities often unstable during the cell culture. Wu et al. employed a special microdevice to fabricate methacrylamide-modified gelatin (GelMA) microfibers with well-defined grooves on their surfaces [55] (Figure 3ci). Compared to the GelMA fibers with smooth surfaces, cells cultured on the resulting fibers allowed a high degree of growth orientation. Groove structures on the fiber surface could be designed using different concentrations and flow rates of GelMA. In general terms, GelMA fibers incorporating grooves showed better cell adhesion and cell encapsulation when compared to microstructured alginate fibers (Figure 3cii,ciii).

Although photopolymerization using microfluidic systems offers a direct route to the preparation of fibers with user-defined structures, the general method is somewhat limited due to strict requirements regarding polymerization kinetics. Additionally, most bioactive molecules including proteins and enzymes are susceptible to UV radiation damage.

### 3.2. Diffusion-Controlled Ionic Cross-Linking

Compared to photopolymerization, ionic cross-linking is more popular for the preparation of biocompatible microfibers under mild conditions. In this method, covalent or noncovalent bonds were formed between pre-polymers and cross-linkers, inducing polymerization. The ionic cross-linkers induce solidification of pre-polymers by the diffusion of ions into the reaction system. In a coaxial flow, solid cylinder microfibers could be formed through setting the pre-polymer as the core flow while setting the ions solution as the sheath flow. Moreover, hollow tubular microfibers could also be fabricated by adding another liquid phase to the core flow, which could be easily removed later while selecting sample solution as middle flow phase. The size of fibers could be well defined by tuning the flow rate ratio between the core and sheath flow.

Among other biomaterials, alginate is a typical type of natural material used in the fabrication of microfibers via diffusion-controlled ionic cross-linking. Based on a coaxial flow-focusing, a sodium alginate solution is usually chosen as the sample core flow, while a CaCl_2_ solution is introduced as the sheath flow to make solid cylinder fibers. This method was first designed by Lee et al. [60], who used a glass capillary combined PDMS chip to prepare calcium alginate fibers in a continuous manner. The introduction of the sheath flow into the system not only provided cross-linkers in a controlled manner, but also prevented microchannels from clogging due to the lubricant effect provided by the sheath flow.

Actually, calcium alginate fibers can only include limited types of cells, because they lack functionalities to recognize special signals on cells. To improve functionality, Lee et al. prepared chitosan-alginate hybrid fibers using a microfluidic device similar to that shown in Figure 3a but without radiation of UV light [56]. They used these hybrid fibers to encapsulate HepG2 cells, and the results showed that these hybrid fibers had a high encapsulation efficiency of cells compared to pure calcium alginate fibers. Moreover, the HepG2 cells encapsulated within hybrid fibers had a longer lifespan, which confirmed that hybrid fibers provide an improved biocompatible environment without losing stable mechanical properties. Roberta et al. reported that they combined alginate with gelatin and urinary bladder matrix (UBM) [57]. The SaOS-2 cells cultured on each fiber showed an elevated viability over 95% after 14 days of culture.

**Figure 4:**
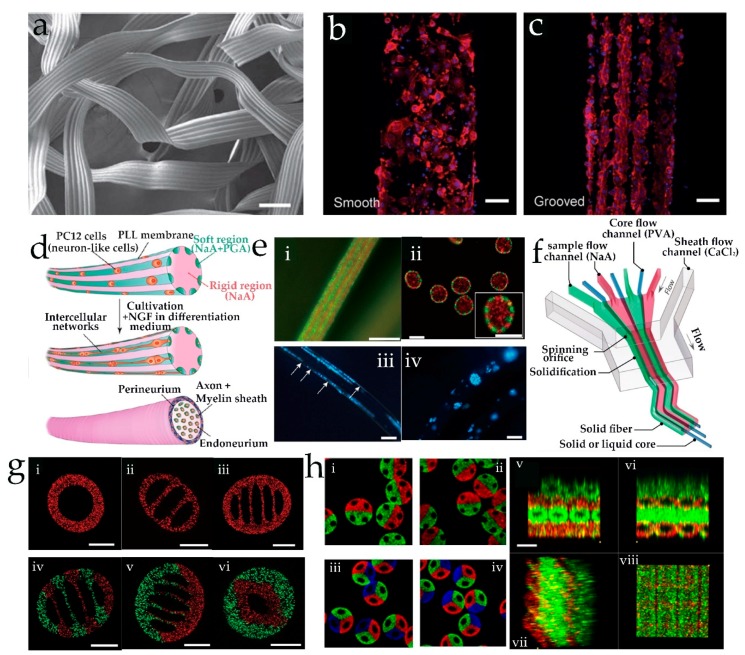
Microfibers with various structures were prepared by microfluidic spinning method. (**a**) Scanning electron microscopy image of flat microfibers with a grooved surface prepared by microfluidic spinning, scale bar: 20 μm; (**b**,**c**) fluorescence microscopy images of myoblasts on flat fibers with smooth surface (**b**) and grooved structures (**c**), scale bar: 50 μm (reprinted with permission from [62]); (**d**,**e**) patterned microfibers for guiding the formation of the network of neural cells, (**d**) complex hydrogel microfiber with rigid regions and cell encapsulating soft regions, (**e**) (**i**,**ii**) fluorescence micrographs of obtained eight-region fiber, (**iii**) cells in the eight-region fibers at 14 days, (**iv**) cells in the homogeneous fibers at 14 days (reprinted with permission from [59]). (**f**–**h**) Multicompartment microfibers manufactured by microfluidic spinning, (**f**) microdevice used to fabricate microfibers with designed hollows, (**g**) cross-sectional (confocal laser scanning microscope(CLSM) image of these hollow microfiber with uniform (**i**–**iii**) and distinctive components (**iv**–**vi**), scale bar: 100 μm (reprinted with permission from [64]); (**h**) cross-sectional CLSM images of Janus microfibers with different compartment and hollow structures (**i**–**iv**), and layer-by-layer architecture by stacking these hollow microfibers (**v**–**viii**); all scale bar indicates 200 μm (reprinted with permission from [58] Copyright (2016) American Chemical Society.).

Flat-shaped alginate microfibers with grooved microstructures were also synthesized by Lee et al. to build scaffolds for cell alignment [62] (Figure 4a–c). The results showed that the cells cultured on groove fibers had rather excellent behaviors of alignment (Figure 4b,c). Unfortunately, the deficiency was that the dimension of grooves was limited by the inner patterns of the microchannel, which suffered from a lack of control flexibility. In order to control chemical composition and morphology of microfibers, a microfluidic chip with special arrangement of channels was employed to synthesize coded alginate microfibers [61]. With this device, microfibers coding with serial, parallel, or mixed heterogeneous composites were continuously prepared. In particular, spatially coded fibers encapsulating different cells were also prepared. Fibers with grooved microstructures and nanoporous spindle-knots could also be fabricated by adjusting the valve operation and changing the inner shape of the microchannels. Combined with the droplet microfluidic technique, peapod-like chitosan microfibers were formed with stable mechanical properties, which exhibited great promise in drugs delivery systems [69]. Normally, the polymerization of alginate was rapid after contact with the Ca^2+^, which forms clog in the microchannels. In order to control the diffusion rate of Ca^2+^, an aqueous buffer flow of polyethylene glycol was sandwiched between the sample flow and the sheath flow [65]. Thus, a continuous production of hollow Ca-alginate fibers was achieved, and the physical and chemical properties of fibers could be adjusted flexibly. These hollow microfibers are unique and delicate templates that are used to build 3D cellular frameworks for 3D complex tissue regeneration. Takeuchi et al. employed a double-coaxial capillary microfluidic reactor to prepare a cellular construct [66]. In this method, a core-shell fiber with cell-encapsulating extracellular matrix (ECM) proteins in the pre-gel state was formed. Next, the cells cultured in the ECM proteins proliferated and differentiated under a stable environment provided by the Ca-alginate shell. In this process, a cellular fiber was formed with desired morphologies and biocompatible functions after the Ca-alginate shell was removed. Different types of cellular fibers were formed with appropriated ECM proteins, which could be folded to form a 3D macroscopic cellular structure. In addition, the prepared islet cell fibers were successfully transplanted into a rat model for the treatment of diabetes mellitus. The results show that these fibers could effectively adjust the glucose concentration in the body of the rat. Unlike the conventional co-axial flow-focusing microdevice, a multilayered poly(methyl methacrylate) (PMMA) microfluidic device was utilized to prepare alginate fibers with a well-defined and complex cross-section shape [59] (Figure 4d,ei,eii). The cross-sectional pattern was dependent on the flow rates of the sample flow and the patterns of the micronozzle array structures. When PC12 cells were induced into the pre-polymer solution, solid hydrogel fibers encapsulating PC12 cells line were obtained in their marginal soft regions after polymerization (Figure 4eiii). Eventually, fibers with intercellular networks on their surface were obtained after a culture period, which were similar to the animal nerve bundles and could be used to build some liner tissues.

Recently, a multiphase parallel co-flow PDMS microdevice was designed by Yu et al. to fabricate hollow fibers with adjustable compartments and heterogeneous ingredients in a single step [64] (Figure 4f,g). It was proved that multicompartment microfibers could be used to synthesize solid polyethylene glycol diacrylate (PEGDA) fibers and culture different cells independently. Simultaneously, Cheng et al. employed a multibarrel capillary microdevice with multiple flows to fabricate alginate Janus hollow microfibers [58] (Figure 4hi–hiv). The inner surface of the hollow fibers could be easily modified with some bioactive materials such as human umbilical vein endothelial cells (HUVECs) and ECM. These fibers could provide an independent 3D environment for different cells and maintain their function. Interestingly, the microfibers encapsulating HUVECs could be woven and stacked into a gridded architecture (Figure 4hv–hviii), which has significant potential for building biomimetic vessels and scaffolds.

In general, alginate is a relatively suitable type of material that is used to fabricate scaffolds for regeneration medicine because of its high biocompatibility, biodegradability, and availability. However, it lacks functionalities to encapsulate cells that require highly specific targeting behaviors of adhesion. Therefore, the synthesis of hybrid fibers with multiple composites is extremely necessary to meet the various requirements of different biological applications.

### 3.3. Solvent Extraction and Other Methods Based on Microfluidic Spinning

Generally, the preparation of microfibers via solvent extraction relies on the diffusion-based mass exchange between the polymer solution and the non-solvent solution. The fabrication of solid poly(lactic-co-glycolic acid) (PLGA) microfibers based on this principle via “on the fly” microfluidic spinning (Figure 3a) was first reported by Lee et al. [70]. In their experiment, the solidification of PLGA was induced by the exchange of dimethyl sulfoxide (DMSO) and water at the interface between the core sample flow and sheath flow. This process, unlike conventional methods, is simple and cost-effective, does not require any complex equipment, and achieves the continuous production of PLGA microfibers with uniform diameters. Recently, hybrid hollow PLGA microfibers were prepared via a co-axial capillary microfluidic device for K^+^-responsive controlled drug release applications [11]. The drug release was controlled by the volume change of encapsulated K^+^-responsive microspheres P(NIPAM-co-AAB15C15). Drugs delivered by those microfibers are considerably appropriated for the treatment of wounds and surgical incisions.

Most conventional methods to synthesize microfibers suffer from the inability to control the dimensions of fibers, as well as the high risk of nozzle clogging. Hydrodynamic microfluidic spinning provides a convenient way to resolve those problems. A simple microfluidic device was designed to prepare poly(methylmethacrylate) (PMMA) fibers through solvent exchange [71]. Because the co-flow channels in this device are 2D-rectangular, diagonal stripe and chevron microstructures are introduced to make the sheath and core sample flow in three dimensions. This device showed flexible control over the size and morphology of PMMA fibers via microfluidic spinning. Due to the ability to precisely manipulate a small volume of liquid, microfluidic spinning could be used to evaluate the effect of reagents on the properties of microfibers. Philippe et al. employed a flow-focusing microfluidic device to analyze the physicochemical properties and proton conductivity of polybenzimidazole (PBI) microfibers [72]. The most important advantage of microfluidic spinning is its ability to synthesize structured microfibers without changing the device structure. Inspired by the spinning process of the silkworm, Lee et al. prepared micro- and nanoscale alginate fibers with relatively ordered structures via dehydration by isopropyl alcohol (IPA) sheath flow on a microfluidic platform [73]. In particular, the nanoscale fibers were fabricated through the induction of Kelvin–Helmholtz instabilities, which was rarely possible to prepare through common microfluidic spinning. In addition, helical dextran microfibers were engineered by a single emulsion microfluidic device [74]. The amplitudes and wavelengths could be flexibly controlled via the flow rate, microchannel dimensions, and concentration of reagents. 

Besides the methods mentioned above, chemical cross-linking [76] and polymerization by heat [77] have also been reported to prepare microfibers with various structures. Specially, biomimetic bamboo-like hybrid microfibers were produced by combining the wet-spinning process with the droplet microfluidic technique [78]. Hydrophobic droplets, PLGA spheres, and mesenchymal stem cells (MSCs) were successfully incorporated into the spherical structure of Ca-alginate fibers. Most importantly, the MSCs encapsulated in such a microstructure showed a high level of viability and proliferation that later formed MSC spheroids, which was an ideal model for the study of in vitro simulation of in vivo growth of microtissues. Microfibers produced in this way exhibited good biocompatibility, as well as enhanced multifunctionality.

To sum up, microfluidic spinning provides a comparably milder reaction condition for preparation of microfibers, which is beneficial for cell encapsulation and bottom-up scaffold fabrication. Heterogeneous materials and complex structures could be flexibly designed by utilizing different microchannels in a mild environment. The microfibers, produced in this manner, demonstrated good mechanical and chemical properties and had great potential to build heterogeneous 3D tissues. Although microfluidic spinning provided quite a few merits compared to other methods, there are still some challenges that need to be addressed. The fibers materials selected for microfluidic spinning are limited because of requirement of short time of solidifying; in addition, their mechanical properties are not strong enough to meet the requirements for the support or regeneration of damaged organs. Therefore, continued efforts are still necessary to fabricate new biocompatible materials with remarkable properties to cater to tissue engineering applications.

## 4. Microparticles/Nanomaterials for Drugs Delivery System

The application of microfluidic chip in traditional synthetic chemistry creates a new instrumental platform that is microfluidics reactor. It is known for the ability of microfluidics technique to efficiently manipulate, process, and analyze chemical reactions on the micrometer even to nanometer scale. The reason why microfluidics route provides unusual advantages over traditional methods is mostly the dependency of microenvironment, which enables the fluid flow characteristic on microscale. First, microfluidic reactor can provide way with which to control mixing and save sample consumption. Second, microfluidic reaction can accelerate chemical reaction and precisely control reactant concentration. Small dimensions of reaction area lower the diffusion times of chemical species, so that mass transport is improved in microfluidic reactors. Third, a high surface-to-volume ratio of microfluidic channel enables heat generated by exothermic reactions to be dissipated rapidly, thereby creating more ‘‘active sites’’ for reactions that are notable for the uniform products. Apart from these microenvironment-dependent advantages, microfluidic design of geometry is also significant in chemical reaction and material synthesis. Typically, the geometry of microfluidic chip can form two manners of fluid flow that are continuous flow and segmented flow. Chemical reaction through both manners meets the merits of efficiently mix reagents, on a short time scale, resulting in a homogeneous reaction environment throughout. In addition to the continuous flow dominated microfluidic reactors, ‘‘segmented flow’’ microfluidic reactors (mostly droplet based segmented flow) are providing alternate approaches to chemical syntheses. Through the possibility of introducing time-control parameters (flow rates and sequence of reagent addition), reactions can be controlled at different stages, resulting in a flexible kinetic control and reactor design according to the reaction mechanism. The main advantage, as well as a challenge, in using microfluidic approaches is the possibility to creatively use these different effects and reaction parameters to produce tailor-made nanomaterials [79].

Microfluidic procedures were found to offer clear advantages over bulk synthesis methods for preparation of microparticles or nanoparticles [11,13,80,81,82] toward biomedicine applications, most notably in the ability to fine-tune the physical properties and chemical compositions of the final product. Meanwhile, microfluidic methods permit in-line detection for monitoring the particles as they form. This not only allows one to study the real-time kinetics of particle growth, but also raises the possibility of using control algorithms to ‘intelligently’ update the reaction conditions and so drive the system towards a desired goal, enabling complete automation of the synthesis procedure.

### 4.1. Nanoparticles for Drugs Delivery System

In recent several years, nanomaterials have been greatly explored in a wild range of biomedical applications, including imaging, catalysis, sensing, and drug delivery. Compared to conventional methods used to fabricate nanomaterials for drugs delivery systems, microfluidics technique has several incomparable features: (1) the particles prepared via microfluidics technique with tunable properties include well-designed particles sizes, structures, and surfaces; (2) unlike most batch-to-batch process with great variation, microfluidics preparation of nanoparticles has high reproducibility and narrow size distribution in a robust way [83]; (3) the nanoparticles prepared in this way with uniform size show precisely controlled payload released behaviors; (4) the nanoparticles fabricated process good versatility, and most of them are water insoluble with soluble drugs loaded; (5) combinatorial preparation of hybrid nanoparticles [84] and high throughput formulation optimization [85] can be easily achieved on microfluidic platform. All the above-mentioned advantages make microfluidics as an ideal technique that will definitely facilitate the implementation of the quality-by-design strategy towards nanoscale drug-delivery systems.

Generally, nanoparticles possess many merits compared with other therapeutics that make them popular in cancer therapy; for example, nanomaterials have increased tumor uptake due to the enhanced permeability and retention effect, targeted delivery to specific tissues with surface conjugated ligands, and systemic circulation times [86]. However, in fact, nanomaterials only reduce the toxicity to bodies compared to conventional chemotherapeutics rather than improved therapeutic efficiency. Improved delivery efficiency is an essential prerequisite of clinical translation of nanoparticles for cancer therapy application [87,88]. Santos et al. employed microfluidics technique to fabricate nanoparticles with core-shell structure to improve the drugs loading efficiency [89]. They selected poorly water-soluble anticancer drug nanocrystals sorafenib as core materials and biodegradable polymer, spermine-functionalized acetalated dextran as shell materials. Through single-step microfluidic nanoprecipitation process, they obtained uniform nano-in-nano vector with higher inhibitory efficiency towards cells compared to conventional vectors due to the high encapsulation of drugs. Subsequently, Santos et al. used similar methods to fabricate core-shell nanocomposites only within milliseconds [90]. However, this time they employed 3D glass capillary device on fabricated nanocomposites in a sequential microfluidic nanoprecipitation. This microfluidics preparation not only maintained high drugs loading efficiency but also achieved high-throughput production so that nanocrystal drugs encapsulated nanocomposites were fabricated at the rate of 700 g/day. In addition, the short interval between the sequential nanoprecipitation ensured stable preparation of nanocomposites without any stabilizer.

Due to complexity of cancer and human immune system, the combination therapy of the co-administration of multiple drugs is desired to generate synergistic therapeutic effects. Thus, the therapeutic aim requires the loading of multiple drugs into a single drugs carrier. However, this is still a challenge to most conventional methods. Microfluidic technique had proved to be an efficient approach with which to prepare composite drugs carrier while simultaneously load several drugs. Porous silicon (PSi) particles are popular as drug carriers because of their biocompatibility, biodegradability, and large surface area and pore volume. Santos et al. fabricated multistage pH-responsive polymer/PSi micro-composites with uniform size for controlled drug release [91]. However, the pores in the Psi are freely accessible, and the drugs loaded in those pores are not well-controlled because of lack of environment protection. Thus, Santos et al. selected a polymer matrix, the acid-degradable acetalated dextran, to seal drug-loaded pores temporarily [92]. The polymer has a tunable, degradable property that helps to control the drug release rate. In addition, those polymers could have modified some functionalities that enhanced the cell uptake. Several drugs were encapsulated into those nanocomposites with a ratiometric control and achieved tunable release.

In a word, microfluidics has a great number of advantages to fabricate nanoparticles, such as flexible manipulation of reagents, synthesis with high reproducibility, and effective control over the physicochemical properties of the prepared nanoparticles. Although microfluidics technique, to some extent, helped to solve the problem of low drugs encapsulation efficiency, there still are some challenges to be solved in the future study, for example, the integration of microfluidics devices needs to be made a priority to achieve automatic production.

### 4.2. Microparticles for Drugs Delivery System

Considerable attention has been directed towards microparticles (MPs), which show nascent utilization in drugs delivery systems. Microparticles serving as therapeutic product carriers must meet following requirements: (a) favorable biocompatibility, (b) high monodispersity, and (c) well-controlled release. In other words, MPs with tailored sized and custom-designed structures are highly desired to improve their performance in drugs delivery systems. The selection of right materials to contain biological molecules or live cells is of high importance. As Kodzius et al. demonstrated, the biomolecules interact with various materials, and the best material can be selected for a given purpose to minimize possible inhibition [93]. It has been found that materials with biocompatible and degradable properties, such as alginate, agarose, PLGA, polylactide microcapsules, PEG, and polylactic acid (PLA) are relatively suitable for those biomedical applications. There are numerous traditional bulk methods for the preparation of MPs, such as mechanical agitation, emulsion polymerization, seeding polymerization, and precipitation. However, these approaches shed light on the limitations of polydispersity and the lack of flexibility in control structures. Microfluidics has increasingly been developed to fabricate MPs with various structures and shapes for different biological applications. Based on this, we can roughly categorize microparticles into microspheres (microgels, microcapsules, and various core-shell structures) and non-spherical microparticles (ellipsoid, Janus particles, and other shapes) according to their shape and structure. Considering the context space, we only focus on the microparticles with potential biological applications, for example, drug delivery. For more extensive details, the reader is referred to number of excellent reviews published elsewhere [94,95,96,97].

#### 4.2.1. Microspheres

**Figure 5:**
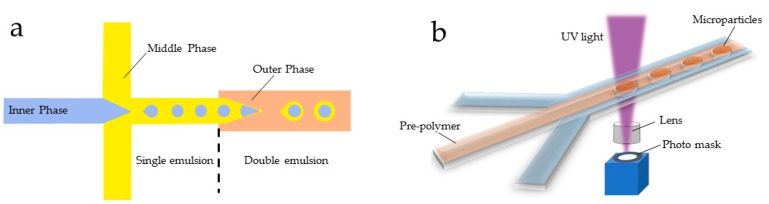
Microfluidic methods used to fabricate microparticles. (**a**) Droplet-based microfluidics and (**b**) flow lithography-based microfluidics.

Microspheres are able to provide high dose drug loading or drug encapsulation efficiency and maintain sustained drug release rate for relatively long periods of time. There are predominantly two means with which to fabricate microspheres using a microfluidic system: one is the droplet-based microfluidic manner and the other is the flow lithography-based microfluidic method (Figure 5).

Microspheres could be generated via droplet-based microfluidic technique. Hussain et al. reported a successful preparation of PLGA-b-PEG microparticles through a flow-focusing microfluidic device, which demonstrated good performances in delivery of therapeutic products [98]. To a certain extent, microfluidic technique has also been employed to fabricate various magnetic microspheres, which had significant role in the controlled release of drugs on the principle of noninvasive magnetic drug targeting [99]. Generally, the size distribution and morphology of microspheres are preferentially considered in the selection of drug carriers, in addition to their low toxicity, high biocompatibility, and biodegradability. Kim et al. [100] used a flow-focusing microfluidic chip to synthesize monodispersed alginate magnetic microspheres containing ultra-small superparamagnetic iron oxide (USPIO) and eluted 6-methoxyethylamino numonafide (MEAN), which were applicable to drug targeting and selective transcatheter drug delivery to the hepatocellular carcinoma.

Microgel is one important type of microspheres. In addition, it has been proved that encapsulating cells in microgels is an promising approach with which to deliver pharmaceutical agents [101]. In fact, most hydrogel particles were fabricated via droplet emulsion template that has an unfavorable influence on viability of cells due to the long exposure to oil environment. To solve this problem, Choi [102] and Lee et al. [103], respectively, fabricated cell-laden microgels through double emulsion drops with a ultra-thin oil shell, which considerably improved the viability of cells. Through changing the hydrophilicity and hydrophobicity of the channel, Li et al. reported a method to synthesize alginate microgels via water-in-oil-in-water emulsions [104]. As we know, the lipophilic compounds are hardly encapsulated into microgel because of the aqueous core of droplet template. However, Mélanie et al. successfully encapsulated lipophilic molecules inside alginate microgels by encapsulating several oil droplets within alginate droplets, which is significant for expanding the application of alginate as drug carriers [105].

Unlike most natural hydrogels, synthetic hydrogels are easily modified with some functional groups to improve their biocompatibility. At the same time, it is easy to achieve a scale-up production due to the well-established polymer chemistry. Polyethylene glycol (PEG) has received great attention due to its low protein absorption, which means it has a small immune response. A large amount of literature has reported fabrication of PEG microgels for medicine treatment, drug delivery, gene test, and so on. Foster et al. [106] produced PEG-4MAL (4-Arm polyethylene glycol-maleimide) in microfluidic chip to promote in vivo vascularization. Torsten et al. used microfluidic cross-flow method to form cell-laden hyperbranched polyglycerol-polyethyleneglycol (hPG-PEG) microgels [107]. As a matter of fact, the hydrophilic nature of the PEG makes it difficult to load hydrophobic drugs into their core. To overcome this, drug-loaded PLGA nanoparticles were incorporated into PEG microgel via droplet-based microfluidics [108]. This hybrid microgel achieved sustained release of hydrophobic drugs.

Microcapsules are another important type of microspheres that normally have a core–shell structure. They have potential application in many fields including food additives [109], agriculture [110,111], microsensors [112,113], cell encapsulation [102,103,114], multiplex assays [115], and the controlled release of drugs in delivery systems [12,116,117,118]. Generally, the shape and size distributions of microcapsules have a considerable influence on their application properties. For example, in the drug delivery application, the distribution of the microcapsules in the animal body and their interplay with cells are significantly affected by the size of the capsules [119]. Microfluidics has been progressively employed to fabricate microcapsules with uniform size distribution, well-defined structure, and narrow polydispersity.

The principle method used to fabricate microcapsules in microfluidic devices is to use droplet emulsion as template. Commonly, single-emulsion droplets or multiple-emulsion droplets are generated through microfluidic flow-focusing device in the first step. Then, a shell solidification process is necessary to maintain the structure of the obtained emulsion. There are mainly four methods with which to solidify the shell as reported: polymerization [113,120,121], evaporation-induced consolidation, freezing, and de-wetting [122]. Among these methods, UV-induced interfacial free radical polymerization seems currently the most popular.

In fact, majority of traditional methods for fabricating microcapsules suffer from the drawbacks of multiple steps and time-consuming. Thus, significant efforts have been made to develop one-step fast formation of microcapsules. Watanabe et al. [123] reported a simple method with which to fabricate monodisperse polylactide microcapsules with an aqueous core via a Y-type microfluidic device by employing the mechanism of spontaneous emulsification and solvent diffusion. Gilad et al. presented a novel and rapid fabrication of polyelectrolyte-based microcapsules in a one-step microfluidic fashion [122]. To be more specific, they utilized the ability of polyelectrolytes to generate complexes across the drop interfaces. Numerous intelligent microcapsules have also been produced for smart drug delivery that exclusively release the inclusion from the core of the microcapsules when exposed to a specific stimulus, such as temperature fluctuation [124,125], pH change [126], certain molecule or ion, external stress, or ultrasonication [127]. By engineering the function of the shell of microcapsules, controlled drug release could be achieved, including sustained release, triggered release, and smart release [12]. In terms of the application of drug carriers, polymer microcapsules have been universally adopted for microencapsulation. Zhou et al. [127] synthesized eccentric and core-centered hollow PDMS microcapsules using microfluidic devices and found that the eccentric microcapsules exhibited a high drug release rate under ultrasonic conditions.

The smart release ability is defined as capsules that can release encapsulates only when exposed to certain conditions. This ability is significant for reducing the side effects of excessive injections for patients. Glucose-responsive microcapsules with a reversible swelling/shrinking behavior under physiological temperature were fabricated by Chu et al. in a glass-capillary microfluidic device [125]. They introduced 3-acrylamidophenylboronic acid (AAPBA) as the glucose sensor, and poly (*N*-isopropylacrylamide) (PNIPAM) as thermo sensor. The resultant microcapsules not only responded to the glucose concentration, but also had a reversible and controlled drug release behavior that is promising for the development of self-regulated delivery systems for diabetes and cancer therapy. However, there might still be some leakage of small molecules encapsulated in the capsules because of the large mesh size of the hydrogel network. Aram et al. introduced grapheneoxide (GO) into the continuous phase, which deposited on both inner and outer interfaces of the microcapsules and greatly lowered the shell permeability [113].

Recently, some work has also focused on tissue regeneration and cell-based therapy. It would be critical to develop in vitro cell cultures in a physiological environment in which cells have a high degree of physiological viability and pluripotency. Microcapsules could provide a stable environment for cell cultures and provided a promising prospect for cell-based medicine research. Embryonic carcinoma cells, human dermal fibroblasts, and mouse embryonic stem cells (ESCs) have been successfully encapsulated within microcapsules prepared via microfluidic devices [114,128,129], which shows a high degree of cell viability and an excellent ability of proliferation and differentiation.

In addition, microcapsules encapsulating colloidal nanomaterials used as biomolecular sensors have aroused interests in the field of biomedicine. It has been proved that microcapsules have preferable sensor performance that encapsulates nanomaterials and avoids biodegradation of organism [115]. This kind of microcapsule could be further immobilized into biocompatible hydrogel to form implantable devices into the human body and achieve real-time monitoring of disease for early-stage disease diagnoses. To realize this, the shell of microcapsules must have the properties of a semipermeable membrane, which only permits the free permeation of biomolecules but prevents the colloidal from diffusing out of the microcapsules.

Nowadays, multiplex assays have been quickly developed to meet the requirement of modern medicine and analytical chemistry at cutting edge. Apart from barcode microbeads, encoded microcapsules are also applicable to multiplex assays. Compared to spectral coding methods, there is absolutely no concern about the spectral overlap problems of using graphical codes on the microcapsules. Kwon et al. encoded the microscale graphical codes on the shell of microcapsules in order to recognize the content encapsulated in the microcapsules [130]. The encode microcapsules were then self-assembly to form microarray. The release of the liquid inside microcapsules was induced through a mechanical releasing system, demonstrating a highly precise and non-damaging release performance.

#### 4.2.2. Non-Spherical Microparticles

In recent years, researchers have shown that the shape of particles can directly influence their biodistribution in vivo, being closely related to their uptake mechanisms and their blood circulation time in the human body [131]. Santos et al. summarized that the physicochemical properties of drugs delivery systems would affect their in vivo or vitro behaviors [80]. For example, particles with largest ratio respect and sharper angle are taken large amount and faster rate. Therefore, research has increasingly focused on the applications of non-spherical microparticles for cell culturing [132], protein encapsulation [133], cell analysis [134], and so on.

Since the first microfluidic method was developed using UV to solidify hydrogels to generate non-spherical microstructures [135,136], large quantities of new methods and technologies have been developed in this field to create a variety of non-spherical micro structures [28,75,137]. These methods include continuous flow lithography (CFL) [138], stop flow lithography (SFL) [139], and a fully-automated SFL compressed-air flow control system [140]. For example, George et al. generated various kinds of monodisperse particles with diverse shapes including scoop, drum-like, and cylindrical shapes using specific factors including UV-light, thermal block, and steric hindrance, manifesting a further potential in the delivery of drugs and therapeutic diagnosis [141]. Wang recently used Janus structure microgels as a template to embed MSCs and HUVECs in microgels at single-cell level [142].

The methods of generating microparticles or nanoparticles with desired shape and structure present a further application in the synthesis of therapeutic products. Thus, it is possible to improve the flexibility of therapeutic products by designing flexible and fine structure to satisfy the increasing physiological demands of tissue engineering in the human body, and these novel materials will have incomparable advantages.

## 5. Discussion and Conclusions

In this review, we discussed the microfluidic synthesis of three types of biomaterials in the field of bioengineering and biomedicine, which include liposomes for artificial cells and organelles, micro-/nanofibers for tissue engineering, and micro-/nanoparticles for drug delivery. It was confirmed that the microfluidic technique shows improved efficiency in biomaterial synthesis compared to conventional bench-top methods in the aspect of particle size, uniformity, stability, structure, and property.

Concerning liposomes, stability and encapsulation efficiency are highly emphasized for delivery systems. Furthermore, specific size and size distribution and function-modified surfaces are also desired for liposomes used to build artificial cells. Usually, the conventional preparation methods suffer from low encapsulation efficiency, polydisperse sizes, and multilamellar structures. The microfluidics technique ensures the reproducible preparation of liposomes with well-defined structures and relatively high monodispersity. In addition, surface modification and functionalization could be easily incorporated into liposome membranes without disrupting the integrity of the membrane. The microfluidic de-wetting process can also improve the expression and transmembrane transport of biomolecules.

Microfibers, as a promising tissue engineering materials, provide the desired properties for 3D cells cultures, which play a crucial role in creating complex tissues in vitro. To prepare microfibers, it generally requires multiple complex postprocess, and presents a high risk of clogging the extrusion, which hinders continuous production. Microfluidic hydrodynamic device can avoid these disadvantages. The sheath flow has a lubricant effect on the wall of microchannels that prevents the clogging of channels. It enables the formation of a well-defined cross-sectional pattern and ensures the active and versatile encapsulation of bioactive molecules into fibers.

The development of advanced materials for drugs delivery systems is another significant part of synthetic biology. During the last few decades, a great number of materials have been developed to achieve smart drug carry and release via microfluidics method. These materials include nano- and microparticles with various structures and properties, for example, microcapsules, Janus particles, microgels, and core-shell microspheres. These materials show well-controlled release rate when exposed to a specific stimulus, such as temperature fluctuation, pH change, chemical signal, and physical stress, or even ultrasonication. Their surface property can be finely modified by bonding a specific protein as an “acceptor” to realize target drug release in tissues or organs, which greatly reduces the side effects of drugs in the human body. Moreover, the uniform particle size also ensures controlled drug dosage and efficient cellular uptake.

To conclude, the microfluidic method offers the advantages of fast mixing, efficient heat and mass exchange, and flexible control of reaction process. It has promising significance for biomaterial synthesis, especially for novel structure and property exploitation as we stated above. However, it has to be noted that microfluidic technology still faces some critical challenges. For example, it is still difficult to realize large scale fabrication, because fluidic control in micron-sized channels is complex. To maintain a stable flow (especially for droplet microfluidics) normally requires surfactants to lower the surface tension of the interface; these added substances run the risk of contaminating the products. Particles may deposit on the inner wall of the small channel, which will accumulate to clog the channel and disrupt the flow continuity.

## Figures and Tables

**Table 1:** Advantages and disadvantages of different methods based on microfluidics used to fabricate liposomes.

Method	Advantages	Disadvantages	Liposomes Diameter	Reference
Electroformation	Simple and rapid	Large size polydisperisity, inapplicable to ionic solutions, low encapsulation efficiency	5–150 μm	[24,25]
Hydration	Electric fields are unnecessary	Large size polydisperisity, products are multilamellar; sensitive to phospholipid type and physical conditions, low encapsulation efficiency	1–10 μm	[24]
Extrusion	Reduced size polydispersity	Relatively complex in operation	130–370 nm	[28]
Microfluidic jetting	Products are unilamellar and of controlled size, encapsulation efficiencies are high	Specialized equipment needed, sensitivity to operational parameters and types of materials used	Above 100 μm	[29]
Microfluidic hydrodynamic-focusing	Products are monodisperse, the size and lamellarity of liposomes are easily controlled, high-throughput production	Low liposome concentration in the end-product	50–300 nm	[30]
Droplet emulsion templates	Polymerosomes can be generated, the size and structure of products can be controlled, high encapsulation efficiencies	Solvent may reside between monolayers	20–200 μm	[31]

**Table 2:** Features of three main methods used in microfluidics spinning used to fabricate microfibers.

Method	Advantage	Disadvantage	Morphologies	Reference
Photopolymerization	Simple and fast	The biomaterials loaded on the fiber are limited because of the ultraviolet (UV) radiation	Solid cylinder	[53]
			Microtube	[53]
			Janus	[54]
			Solid cylinder with grooved structures	[55]
Diffusion-Controlled Ionic Cross-linking	Reaction processing under mild conditions, more flexible control of structure of fibers, a wider range of biomaterials could be encapsulated loaded or within fibers	The reaction is affected by diffusion rate of ionic cross-linking agent	Solid cylinder	[56,57,58,59]
			Spiral curls	[60]
			Solid fiber with spindle-knots	[61]
			Tubuliform fibers with nanogrooves	[61]
			Flat microfibers	[62]
			Hollow fibers	[58,63,64,65]
			Core–shell microfibers	[66] [67,68]
			Straight, folded, and coiled structure	
			Cylinder with peapod-like internals	[69]
Solvent extraction	Reaction processing under mild conditions	Limit type of materials used to fabricate fibers because of special requirement of solvent	Solid cylinder	[70,71,72]
			Ribbon-shaped fiber	[71]
			Silk structures	[73]
			Helical microfibers	[74]
			Janus hollow microfiber	[11]
